# Atherosclerosis in 16th-Century Greenlandic Inuit Mummies

**DOI:** 10.1001/jamanetworkopen.2019.18270

**Published:** 2019-12-27

**Authors:** L. Samuel Wann, Jagat Narula, Ron Blankstein, Randall C. Thompson, Bruno Frohlich, Caleb E. Finch, Gregory S. Thomas

**Affiliations:** 1 Ascension Healthcare, Milwaukee, Wisconsin; 2 Icahn School of Medicine at Mount Sinai, New York, New York; 3 Brigham and Women’s Hospital, Boston, Massachusetts; 4 St Luke’s Mid-America Heart Institute, Kansas City, Missouri; 5 Smithsonian Institution, Washington, DC; 6 University of Southern California, Los Angeles; 7 Memorial Care Heart & Vascular Institute, Fountain Valley, California

## Abstract

This case series examines 4 Greenlandic Inuit mummies from approximately the 16th century for evidence of atherosclerosis.

## Introduction

Atherosclerosis is often thought of as unique to modern *Homo sapiens*, the product of our contemporary diet, lifestyle, and environment superimposed on primordial susceptibility. However, the HORUS Study Group has found that atherosclerosis existed at least as far back as 4000 bce.^1^ Arterial calcification has been found in 34 of 137 mummified remains from 3 continents across wide variations in lifestyle and heritage, including in hunter-gatherer populations.^1,2^ None of these individuals consumed a primarily marine-based diet rich in ω-3 fatty acids. Fifty years ago, Danish researchers^3^ hypothesized that high intake of marine animals rich in fish oil containing ω-3 fatty acids protected native Greenlandic Inuit peoples from atherosclerosis. Davis and colleagues^4^ found fish oil reduced the atherosclerosis induced in rhesus monkeys exposed to a high-cholesterol atherogenic diet. In 2019,^5^ interest persists in the actions of ω-3 fatty acids in their natural and highly purified forms. To better understand the early history of human atherosclerosis, we performed a case series study of Inuit hunter-gatherer people living 500 years ago who consumed a marine-based diet.

## Methods

Five Inuit mummies curated at the Peabody Museum of Archaeology and Ethnology, Cambridge, Massachusetts, were studied at the Heart and Vascular Center of the Brigham and Women’s Hospital, Boston, Massachusetts. Permission to perform imaging was granted by the Peabody Museum of Archaeology and Ethnology. These natural mummies, preserved primarily by the cold environment, were discovered by Martin Luther on the Greenlandic island of Uunartaq, Greenland, in 1929.^6^ Grave goods and typical clothing indicated burial in the 1500s, when these individuals would have lived in stone, whale bone, and seal skin huts and hunted from kayaks with spears, bows, and arrows for their diet of fish, birds, marine mammals, and caribou.

Multidetector whole-body computed tomography (CT) images were obtained at 80 and 120 kV with 6-mm slice thickness with 50% overlap and reconstructed using multiple kernels with a third-generation dual-source CT scanner (Siemens). Images were reviewed and interpreted by consensus of 5 cardiologists (L.S.W., J.N., R.B., R.C.T., and G.S.T.) and 2 radiologists with extensive experience interpreting mummy CT images. Age and sex were estimated from bone and dental development by a physical anthropologist (B.F.).

## Results

**Figure 1.**
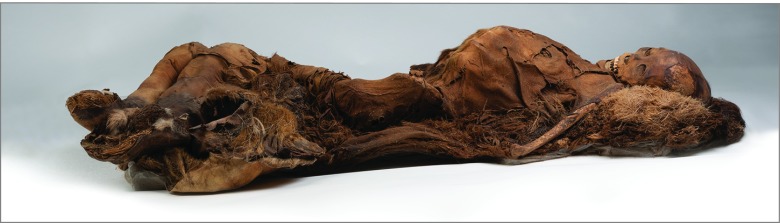
Adult Inuit Mummified Individual Who Was Scanned With Computed Tomography Courtesy of the Peabody Museum of Archeology and Ethnology, Harvard University, PM 29-10-10/61570.0.

**Figure 2.**
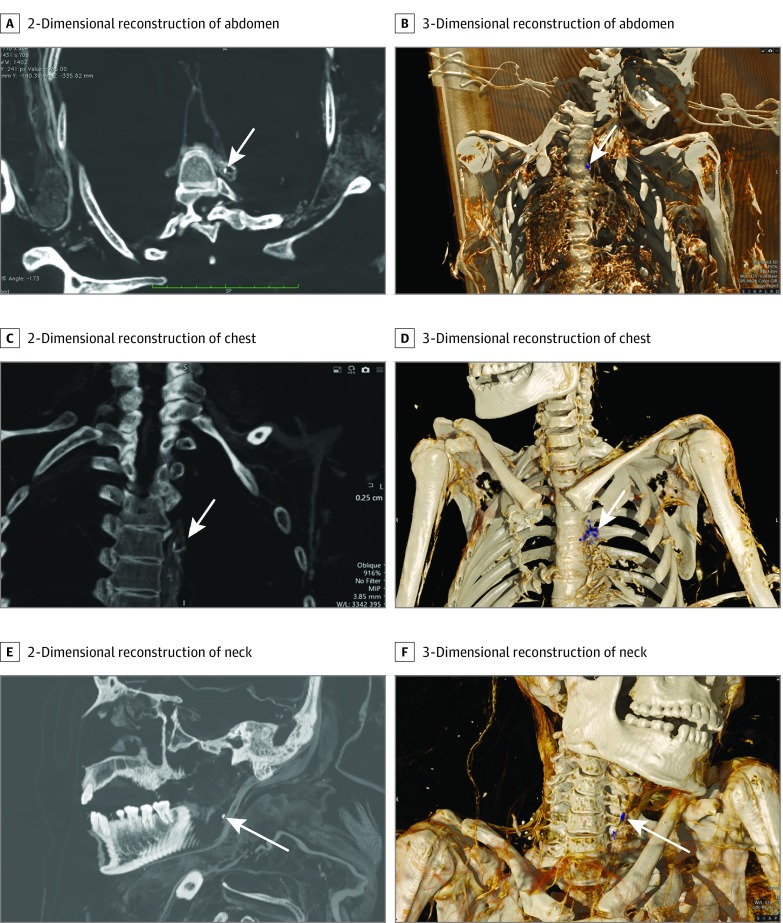
Computed Tomography Images Showing Calcified Atherosclerotic Plaques Arrows indicate calcified atherosclerotic plaques.

An infant mummy was excluded from further analysis owing to paucity of non–bony tissue. Based on skeletal and dental features, the remaining mummies were adolescents or young adults, including 2 men who died at ages 18 to 22 years and 25 to 30 years and 2 women who died at ages 16 to 18 years and 25 to 30 years (Figure 1). The causes of death could not be determined. Remnants of the carotid arteries, the thoracic and retroperitoneal aorta, and iliac arteries were preserved in all 4 individuals, but reliable anatomic landmarks within the heart could not be identified. Three mummies had evidence of calcified atheroma, identified as discrete high-density regions in an arterial distribution (Figure 2). Incomplete visualization of the arterial vascular tree precluded accurate grading of the magnitude or severity of vascular calculation and evaluation of clinical disease. Nevertheless, the appearance of vascular calcification in these 3 mummies resembled previous observations of atherosclerosis in mummies and living humans.

## Discussion

This cases series presents evidence for the presence of calcified plaques in the mummified remains of 3 young Inuit individuals living 500 years ago, suggesting the presence of atherosclerosis despite their vigorous lifestyle and marine-based diet. While we cannot know the incidence of ancient ischemic events, cardiovascular deaths were rare among mid-20th century Inuit people, similar to contemporary Amazonian Tsimane people, who have low-grade atherosclerosis and low incidence of cardiovascular death.^7,8,9^ The etiologic complexity of atherosclerosis confounds identification of single factors, such as ω-3 fatty acids, as causal or protective. Other factors may include environmental smoke,^10^ which is produced by indoor fires used by Inuit and many other ancient peoples who also incurred atherosclerosis.
