# Addendum to Figure Caption

**DOI:** 10.1001/jamanetworkopen.2020.0165

**Published:** 2020-01-22

In the Research Letter titled “Atherosclerosis in 16th-Century Greenlandic Inuit Mummies,”^1^ published December 27, 2019, the following should have been included in the caption of Figure 1: “This Figure should not be reproduced without permission of the Peabody Museum of Archaeology and Ethnology at Harvard University. Requests should be sent to pmimages@fas.harvard.edu.” This article has been corrected.^1^
